# First Detection and Molecular Characterization of Usutu Virus in *Culex pipiens* Mosquitoes Collected in Romania

**DOI:** 10.3390/microorganisms11030684

**Published:** 2023-03-07

**Authors:** Florian Liviu Prioteasa, Sorin Dinu, Georgiana Victorița Tiron, Ioana Georgeta Stancu, Elena Fălcuță, Cornelia Svetlana Ceianu, Ani Ioana Cotar

**Affiliations:** 1 Medical Entomology Laboratory, Cantacuzino National Military Medical Institute for Research and Development, 103 Splaiul Independenței, 050096 Bucharest, Romania; 2 Molecular Epidemiology for Communicable Diseases Laboratory, Cantacuzino National Military Medical Institute for Research and Development, 103 Splaiul Independenței, 050096 Bucharest, Romania; 3 Vector-Borne Infections Laboratory, Cantacuzino National Military Medical Institute for Research and Development, 103 Splaiul Independenței, 050096 Bucharest, Romania; 4 Department of Microbiology, Faculty of Biology, University of Bucharest, 1–3 Aleea Portocalelor, 060101 Bucharest, Romania; 5 Department of Genetics, Faculty of Biology, University of Bucharest, 1–3 Aleea Portocalelor, 060101 Bucharest, Romania

**Keywords:** Usutu virus, CDC light trap, CDC gravid trap, BG-Sentinel trap, *Culex pipiens* mosquitoes, phylogenetic analysis, Europe 2 lineage, Romania

## Abstract

Usutu virus (USUV) is an emergent arbovirus in Europe causing mortality in bird populations. Similar to West Nile virus (WNV), USUV is maintained in sylvatic cycles between mosquito vectors and bird reservoirs. Spillover events may result in human neurological infection cases. Apart from indirect evidence provided by a recent serological study in wild birds, the circulation of USUV in Romania was not assessed. We aimed to detect and molecular characterize USUV circulating in mosquito vectors collected in South-Eastern Romania—a well-known WNV endemic region—during four transmission seasons. Mosquitoes were collected from Bucharest metropolitan area and Danube Delta, pooled, and screened by real-time RT-PCR for USUV. Partial genomic sequences were obtained and used for phylogeny. USUV was detected in *Culex pipiens* s.l. female mosquitoes collected in Bucharest, in 2019. The virus belonged to Europe 2 lineage, sub-lineage EU2-A. Phylogenetic analysis revealed high similarity with isolates infecting mosquito vectors, birds, and humans in Europe starting with 2009, all sharing common origin in Northern Italy. To our knowledge, this is the first study characterizing a strain of USUV circulating in Romania.

## 1. Introduction

Usutu virus (USUV) is a single-stranded, positive-sense RNA virus, belonging to *Flavivirus* genus, *Flaviviridae* family. Similar to other flaviviruses, this emerging pathogen is maintained in sylvatic cycles between ornithophilic mosquitoes as vectors and birds as amplifying hosts. Mammals, including humans, are considered “dead-end” hosts, as they can be incidentally infected through mosquito biting, but do not develop a sufficient viremia to sustain transmission [1,2].

USUV was first isolated in 1959 from *Culex neavei* mosquitoes collected in South Africa [3]. In Europe, USUV was first detected in Austria, in 2001, when it produced increased mortality in wild birds in Vienna [4]. However, retrospective study based on archival bird tissues presented evidence of a much earlier circulation of the same strain in 1996, in Tuscany, Italy [5]. Since the first report, the virus was confirmed as cause of mortality in wild and captive birds in Europe, especially in *Passeriformes* (e.g., common blackbirds—*Turdus merula*) and *Strigiformes* populations [6,7,8,9]. As reviewed [2], virus isolation, nucleic acid detection, and seroconversion studies detected USUV in a wide range of other vertebrates in Europe, such as horses, dogs, bats, squirrels, wild boars, roe deer, bats, and lizards. Similar to the West Nile virus (WNV) transmission cycle, *Culex pipiens* mosquitoes act as vectors of USUV in Europe [10], although invasive species such *Aedes albopictus* [11] and *Aedes japonicus* [12] were also found infected.

Worldwide diversity of USUV is reflected in the existence of eight genetic lineages (Africa 1–3 and Europe 1–5) [13,14]. Despite being recently detected in Europe, phylogeny study concluded that USUV was introduced regularly from Africa into Europe in the last 50 years [13]. Co-circulation of both African and European lineages was documented in Europe or sometimes even in the same country. However, Europe 2 lineage is considered to be the most prevalent there [2].

To date, only approximately one hundred symptomatic and asymptomatic USUV human infection cases were recorded globally, mostly in Europe. Thirty of those cases were neuroinvasive infections [15].

Seroprevalence study on wild birds conducted in South-Eastern Romania between 2018 and 2019 presented indirect evidence of USUV circulation in this country [16]. However, serological survey conducted between 2019 and 2020 on healthy blood donors showed no evidence of USUV circulation in human population from the North-Western region of Romania [17].

Knowledge on USUV circulation in Romania is extremely scarce and there is no sequence data regarding the circulating strains. To overcome these gaps, we set up an entomological study aiming to characterize the USUV detected in pools of mosquitoes collected in Bucharest and Danube Delta, during four transmission seasons.

## 2. Materials and Methods

### 2.1. Sampling Sites and Mosquito Collection

**Figure 1:**
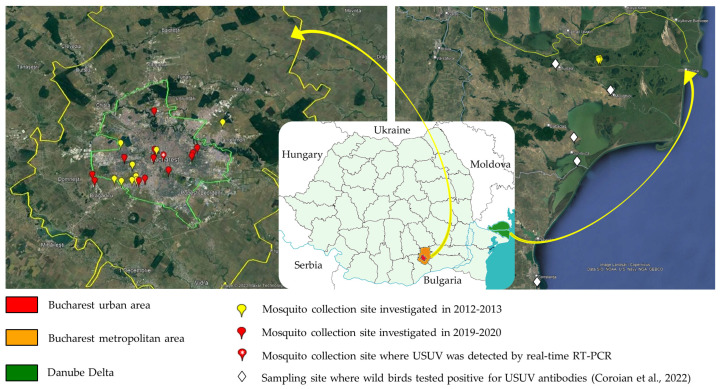
Mosquito sampling sites, South-Eastern Romania, 2012–2013 and 2019–2020. Sampling sites where wild birds tested positive for USUV antibodies were reported by Coroian et al. [16].

Adult mosquitoes were collected each year between July and September. Nine sites located in Bucharest metropolitan area and four sites located in Danube Delta (the left bank of the Sulina branch, opposite to Vulturu village, Tulcea county) were investigated in 2012 and 2013. During 2019 and 2020, the study was carried out in thirteen sites located in Bucharest metropolitan area (Figure 1). Collections were performed using Centers for Disease Control and Prevention (CDC) light and gravid traps (John W. Hock Company, Gainesville, FL, USA), BG-Sentinel traps (Biogents, Regensburg, Germany), bird-baited traps, and mammal-baited traps. Chickens and guinea pigs were used as baits after obtaining the approval of Cantacuzino Institute Ethics Committee. Animal-baited traps were used only in 2012–2013 seasons in the Danube Delta. CDC light traps and BG-Sentinel traps were used in 2012–2013 seasons both in Danube Delta and Bucharest metropolitan area. In 2019–2020 seasons we exclusively used CDC gravid traps. All these traps proved good collection rates and can operate continuously for several days. CDC gravid traps attract mainly pregnant mosquito females, thus increasing the chances of finding arbovirus positive samples. Mosquitoes were transported to the laboratory in liquid nitrogen containers and there were kept at −70 °C freezer until further processing.

### 2.2. Mosquito Processing and RNA Extraction

Mosquitoes were identified to species level on chill table under stereomicroscope, using dichotomous keys described by Becker et al. [18] and grouped according to species, sex, physiological age, site and date of collection, into pools never exceeding 50 individuals. Viral RNA was extracted from pool homogenates using QIAamp Viral RNA Mini Kit (Qiagen, Hilden, Germany), as previously described [19].

### 2.3. Detection of USUV by Real-Time RT-PCR

Mosquito samples collected between 2012 and 2013 were tested using primers and probe described by Del Amo et al. [20] and GoTaq^®^ Probe 1-Step RT-qPCR System (Promega, Madison, WI, USA). Briefly, reaction mix contained five microliters of RNA, 0.25 µM of each primer, 0.2 µM probe, 0.4 µL GoScript™ RT Mix for 1-Step RT-qPCR, ten µL of GoTaq^®^ Probe qPCR Master Mix with dUTP, and RNAse-free water up to 20 µL. Thermal amplification was carried out following the profile recommended by the real-time RT-PCR kit manufacturer on an Mx3005P Real-Time PCR System (La Jolla, CA, USA). For testing the samples collected between 2019 and 2020 a real-time RT-PCR assay developed by Nikolay et al. [21] and SuperScript™ III Platinum™ One-Step qRT-PCR Kit (Invitrogen, Waltham, MA, USA) were used. Primers and probe concentrations were 0.5 µM and 0.2 µM, respectively. A total of 10 microliters of RNA extract were tested in a final reaction volume of 25 µL. Thermal amplification was carried out following the profile recommended by the real-time RT-PCR kit manufacturer on an Mx3005P Real-Time PCR System (La Jolla, CA, USA). Each PCR run was validated by including a positive control (USUV strain SAAR-1776, 1959, GenBank acc. no. AY453412) and a negative control (PCR grade water).

### 2.4. Sequencing and Phylogenetic Analysis

A primer walking strategy [22] was employed for generating amplicons spanning genomic regions preM-M-E and NS4B-NS5-3′UTR, respectively. PCRs were carried out using five microliters of RNA extract, 0.8 µM of each primer, and OneStep RT-PCR Kit (Qiagen, Germany) according to the kit insert. Amplicons were sequenced with BigDye™ Terminator v3.1 Cycle Sequencing Kit on a SeqStudio™ Genetic Analyzer (Applied Biosystems, Waltham, MA, USA). Consensus sequences were obtained with BioEdit version 7.2.5 [23]. Phylogenetic trees were generated using MEGA11 version 11.0.13 [24] after choosing the fittest nucleotide substitution model with the same software. The tree annotations were added using EvolView v2 [25].

## 3. Results

A total number of 13,823 mosquitoes were collected during the study. Urban collections comprised specimens belonging to *C. pipiens* s.l. and invasive *A. albopictus* species, whereas Danube Delta collections contained specimens belonging to four species (Table 1).

A total of 419 mosquito pools were screened by real-time RT-PCR for USUV. Only one pool yielded positive result (Ct 24). The calculated minimum infection rate—MIR ([number of positive pools/total specimens tested] × 1000)—was 0.19‰. The pool was made up of 17 *C. pipiens* s.l. females, collected in Bucharest urban area during the mosquito season, 28–30 July 2019 (Table 1, Figure 1).

**Figure 2:**
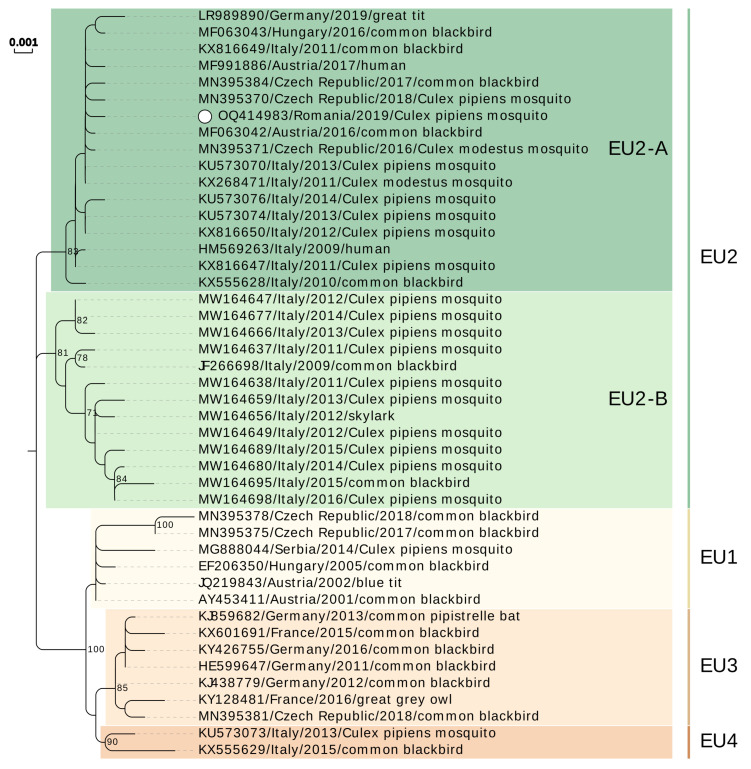
Phylogenetic tree of Usutu virus Europe 1–4 lineages (EU1-EU4) showing the Romanian sequence clustering into sub-lineage EU2-A. White dot: sequence obtained in this study. Lineages were assigned according to [13,14,22,26] and similar sequences retrieved by NCBI BLAST were also included in the analysis. The evolutionary history was inferred from a preM-M-E fragment (nucleotides 591–2447, reference sequence GenBank acc. no. NC_006551) by using the Maximum Likelihood method and Kimura 2-parameter model, 1000 bootstrap replicates. A discrete Gamma distribution was used to model evolutionary rate differences among sites (5 categories (+G, parameter = 0.1000)). Numbers at nodes represent the bootstrap percentages (values <70% are not shown).

**Figure 3:**
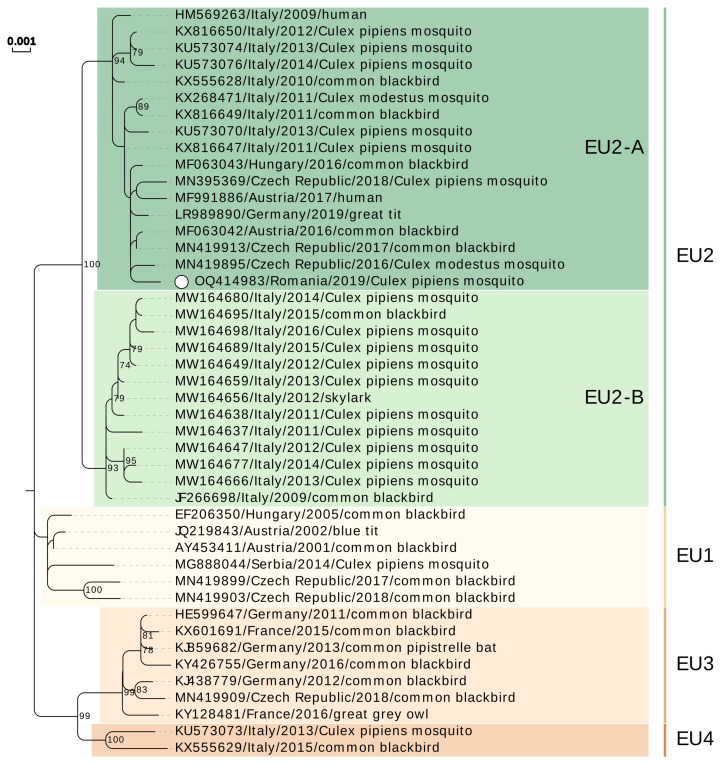
Phylogenetic tree of Usutu virus Europe 1–4 lineages (EU1-EU4) showing the Romanian sequence clustering into sub-lineage EU2-A. White dot: sequence obtained in this study. Lineages were assigned according to [13,14,22,26] and similar sequences retrieved by NCBI BLAST were also included in the analysis. The evolutionary history was inferred from a NS4B-NS5 fragment (nucleotides 7372–10,398, reference sequence GenBank acc. no. NC_006551) by using the Maximum Likelihood method and Tamura–Nei model, 1000 bootstrap replicates. A discrete Gamma distribution was used to model evolutionary rate differences among sites (5 categories (+G, parameter = 0.1000)). Numbers at nodes represent the bootstrap percentages (values <70% are not shown).

First partial genomic sequence (1857 nt) contained almost the entire coding sequence of preM, whole coding sequence of M protein, and partial E protein (nucleotide positions 591–2447, reference sequence GenBank acc. no. NC_006551). The second genomic fragment (3154 nt) spanned positions 7356–10,509 and contained partial coding sequence of NS4B protein, entire coding sequence of NS5 viral polymerase, and partial 3′UTR. The two sequences were deposited in GenBank as a gapped format submission under the acc. no. OQ414983. The two sequences were used for assigning genetic lineage of the detected USUV [13,14,22,26]. Phylogenetic analyses placed the USUV detected in this study into Europe 2 lineage, group A/sub-lineage EU2-A. Romanian sequences clustered with USUV sequences obtained from *Culex* sp. mosquito vectors, birds (common blackbird and great tit), and human samples collected starting with 2009 in Italy, Central Europe (Austria, Czech Republic, and Hungary), and Germany. The topology of the two phylogenetic trees constructed was consistent (Figure 2 and Figure 3).

We investigated our sequences for the presence of phylogenetic informative substitutions (i.e., cluster or country specific substitutions) [13,26], host specific mutations [13], and markers linked to neuroinvasiveness [27]. Substitutions G595S and E3425D specific for EU2-A sub-lineage and associated with neuroinvasiveness were identified in Romanian sequences. M2645I substitution characteristic to Italian isolates was also detected.

## 4. Discussion

In this study we present the molecular characterization of USUV detected in mosquito vectors collected in South-Eastern Romania. To our knowledge, this is the first sequence-based evidence of USUV circulation in our country. However, seroprevalence study showed the circulation of USUV in wild birds sampled in localities in Tulcea county, including Danube Delta Biosphere Reserve, and in Constanta county [16] (Figure 1), but not in human population in other part of the country [17]. The sampling sites chosen for our four-year study were Danube Delta and Bucharest metropolitan area. Danube Delta is the largest wetland in Europe and a major stopover for wild birds migrating between Europe and Africa. It was suggested that the migratory birds might act as potential long-distance dispersal vehicles of USUV and that the bird flyways could predict the continental and intercontinental dispersal patterns of this virus [13]. Moreover, Danube Delta was shown to be an area of circulation and introduction for WNV which displays common ecology and epidemiology traits with USUV [28,29,30,31]. Bucharest, the second sampling region in our study, is also known for the intense circulation and human transmission of WNV, significant outbreaks occurring there in the last three decades [32,33,34], with *C. pipiens* mosquitoes acting as vectors [29,35,36].

We detected USUV by real-time RT-PCR in *C. pipiens* s.l. female pool collected in 2019, in Bucharest. Our phylogenetic analyses showed that the virus belong to Europe 2 lineage (EU2). As reviewed elsewhere [2,37], this lineage is the most prevalent on our continent and circulates or co-circulates with other European and African lineages in countries from Central Europe (Austria, Hungary, Czech Republic, and Slovakia), Western Europe (France and Germany) and Southern Europe (Serbia and Croatia). Large scale studies aiming to reconstruct the evolutionary history of USUV have shown that European lineages share a common ancestor and are the result of local evolution [13] and placed the origin of EU2 between 1993 [13] and 2003 [26]. Italy is considered a hot spot for USUV evolution and a dispersal source of European lineages throughout the continent. Recent study on the Italian USUV isolates demonstrated that the EU2 can be split in two groups/sub-lineages, EU2-A and EU2-B, respectively [26]. Our sequence clusters within sub-lineage EU2-A which is considered to have emerged in February 2007 and contains samples collected between 2009 and 2018 from Northern and Central Italian regions, Austria, and Hungary [26].

Specific mutations have been described for the European lineages and sub-lineages. G595S (E protein) and E3425D (NS5 protein) substitutions are considered EU2-A amino acid signatures [26] and are present in the Romanian sequence, too. Substitution G595S was also considered a hallmark of human isolates [13] and along with E3425D was first detected in the viral isolate infecting the first human patient presenting neurological symptoms. G595S substitution is located in DIII domain of E protein that is recognized by neutralizing antibodies. It was hypothesized that the two substitutions play a role in the neuroinvasive capacity of USUV [27]. The relatedness of the Romanian sequence with the Italian cluster is indicated also by the amino acid substitution M2645I (NS5 protein) considered characteristic for the Italian isolates [26].

It was demonstrated that USUV spread in Europe was from Northern Italy to Austria and from there to Hungary and subsequently to Serbia. The study proposes bird populations migration—namely of *Turdus* spp.—as mechanism for virus dispersal across the continent [26]. Due to the high similarity between the Romanian and the cluster of sequences originating in Italy, we can assume that a potential spread from neighboring Hungary or Serbia could have been the source of USUV in Romania.

Although we could not find evidence of USUV circulation in mosquito vectors collected between 2012 and 2013 in Danube Delta, a latter seroprevalence study (2018–2019) documented the presence of USUV in wild birds sampled in the area, mainly belonging to *Passeriformes* and *Bucerotiformes* orders. Of note, the species presenting the highest seroprevalence in this study was the common blackbird [16]. However, the same study failed to detect USUV specific antibodies in birds sampled in Bucharest in the same period when the *C. pipiens* s.l. mosquitoes were found positive in our study, most probably because of the limited number of sera tested.

Only one out of the 419 mosquito pool tested in our study was found positive for USUV in four transmission seasons. USUV might be a poor competitor in this WNV endemic region. The two viruses are antigenically related and the antibodies against WNV may cross-react and prevent USUV infection in reservoir birds previously exposed to WNV, as it was shown in vaccinated mice [38]. As well, it was shown in a laboratory study that WNV is selectively transmitted by *C. pipiens* mosquitoes after being co-exposed to both viruses via an infectious blood meal [39]. The circulation of WNV in 2019, the year when the USUV positive pool was detected, was documented in the Bucharest metropolitan area by 15 confirmed WNV human infection cases [40].

The current available data, although limited, suggested that the level of USUV circulation in South-Eastern Romania was low and might have been overwhelmed by the very active transmission of WNV, which has been causing significant human outbreaks in this region. However, as shown for WNV, in the eco-epidemiological conditions in Romania the emergence of a mosquito-borne virus may occur within a transmission season [36]. Therefore, mosquito and bird surveillance, blood donor screening, and increased awareness regarding the possible involvement of USUV in neuroinvasive infection cases are permanently needed.

## 5. Conclusions

In this study we present the first sequence-based evidence for USUV circulation in Romania. The detected strain belonged to Europe 2 lineage, EU2-A sub-lineage, clustering with viruses originating from Northern Italy, Central Europe, and Germany.

## Figures and Tables

**Table 1:** Mosquito pools screened by real-time RT-PCR for the presence of Usutu virus, South-Eastern Romania, 2012–2013 and 2019–2020.

Collection Area	Mosquito Species	2012	2013	2019	2020	Total per Species
		Number of Mosquitoes Collected/Number of Pools				
Bucharest	*Culex pipiens* s.l.	1791/53	1904/56	5053/139 *	1785/49	10,533/297
	*Aedes albopictius*	0/0	0/0	59/2	0/0	59/2
Danube Delta	*Culex pipiens*	1201/44	1616/58	0/0	0/0	2817/102
	*Culex modestus*	236/11	135/5	0/0	0/0	371/16
	*Anopheles hyrcanus*	0/0	13/1	0/0	0/0	13/1
	*Anopheles maculipennis*	0/0	30/1	0/0	0/0	30/1
Total per study						13,823/419

* One mosquito pool tested positive for Usutu virus in the real-time RT-PCR assay.

## Data Availability

Data can be provided on request from the corresponding author.

## References

[1] Zannoli S., Sambri V. (2019). West Nile Virus and Usutu Virus Co-Circulation in Europe: Epidemiology and Implications. Microorganisms.

[2] Vilibic-Cavlek T., Petrovic T., Savic V., Barbic L., Tabain I., Stevanovic V., Klobucar A., Mrzljak A., Ilic M., Bogdanic M. (2020). Epidemiology of Usutu Virus: The European Scenario. Pathogens.

[3] Williams M.C., Simpson D.I.H., Haddow A.J., Knight E.M. (1964). The Isolation of West Nile Virus from Man and of Usutu Virus from the Bird-Biting Mosquito *Mansonia Aurites*(Theobald) in the Entebbe Area of Uganda. Ann. Trop. Med. Parasitol..

[4] Weissenböck H., Kolodziejek J., Url A., Lussy H., Rebel-Bauder B., Nowotny N. (2002). Emergence of *Usutu virus*, an African Mosquito-Borne *Flavivirus* of the Japanese Encephalitis Virus Group, Central Europe. Emerg. Infect. Dis..

[5] Weissenböck H., Bakonyi T., Rossi G., Mani P., Nowotny N. (2013). Usutu Virus, Italy, 1996. Emerg. Infect. Dis..

[6] Bakonyi T., Erdélyi K., Ursu K., Ferenczi E., Csorgo T., Lussy H., Chvala S., Bukovsky C., Meister T., Weissenböck H. (2007). Emergence of Usutu Virus in Hungary. J. Clin. Microbiol..

[7] Steinmetz H.W., Bakonyi T., Weissenböck H., Hatt J.-M., Eulenberger U., Robert N., Hoop R., Nowotny N. (2011). Emergence and establishment of Usutu virus infection in wild and captive avian species in and around Zurich, Switzerland—Genomic and pathologic comparison to other central European outbreaks. Veter- Microbiol..

[8] Manarolla G., Bakonyi T., Gallazzi D., Crosta L., Weissenböck H., Dorrestein G., Nowotny N. (2010). Usutu virus in wild birds in northern Italy. Veter- Microbiol..

[9] Ziegler U., Bergmann F., Fischer D., Müller K., Holicki C.M., Sadeghi B., Sieg M., Keller M., Schwehn R., Reuschel M. (2022). Spread of West Nile Virus and Usutu Virus in the German Bird Population, 2019–2020. Microorganisms.

[10] Fros J.J., Miesen P., Vogels C.B., Gaibani P., Sambri V., Martina B.E., Koenraadt C.J., van Rij R.P., Vlak J.M., Takken W. (2015). Comparative Usutu and West Nile virus transmission potential by local Culex pipiens mosquitoes in north-western Europe. One Health.

[11] Klobucar A., Benic N., Krajcar D., Kosanovic-Licina M.L., Tesic V., Merdic E., Vrucina I., Savić V., Barbic L., Stevanovic V. (2016). An overview of mosquitoes and emerging arboviral infections in the Zagreb area, Croatia. J. Infect. Dev. Ctries..

[12] Camp J.V., Kolodziejek J., Nowotny N. (2019). Targeted surveillance reveals native and invasive mosquito species infected with Usutu virus. Parasites Vectors.

[13] Engel D., Jöst H., Wink M., Börstler J., Bosch S., Garigliany M.-M., Jöst A., Czajka C., Lühken R., Ziegler U. (2016). Reconstruction of the Evolutionary History and Dispersal of Usutu Virus, a Neglected Emerging Arbovirus in Europe and Africa. Mbio.

[14] Cadar D., Lühken R., van der Jeugd H., Garigliany M., Ziegler U., Keller M., Lahoreau J., Lachmann L., Becker N., Kik M. (2017). Widespread activity of multiple lineages of Usutu virus, western Europe, 2016. Eurosurveillance.

[15] Cadar D., Simonin Y. (2022). Human Usutu Virus Infections in Europe: A New Risk on Horizon?. Viruses.

[16] Coroian M., Silaghi C., Tews B.A., Baltag E., Marinov M., Alexe V., Kalmár Z., Cintia H., Lupșe M.S., Mihalca A.D. (2022). Serological Survey of Mosquito-Borne Arboviruses in Wild Birds from Important Migratory Hotspots in Romania. Pathogens.

[17] Coroian M., Mihalca A.D., Dobler G., Euringer K., Girl P., Borșan S.-D., Kalmár Z., Briciu V.T., Flonta M., Topan A. (2022). Seroprevalence Rates against West Nile, Usutu, and Tick-Borne Encephalitis Viruses in Blood-Donors from North-Western Romania. Int. J. Environ. Res. Public Heal..

[18] Becker N., Petric D., Zgomba M., Boase C., Madon M., Dahl C., Kaiser A. (2010). Mosquitoes and their Control.

[19] Prioteasa F.L., Tiron G.V., Dinu S., Fălcuţă E. (2022). Using Rapid Analyte Measurement Platform (RAMP) as a Tool for an Early Warning System Assessing West Nile Virus Epidemiological Risk in Bucharest, Romania. Trop. Med. Infect. Dis..

[20] Del Amo J., Sotelo E., Fernández-Pinero J., Gallardo C., Llorente F., Agüero M., Jiménez-Clavero M.A. (2013). A novel quantitative multiplex real-time RT-PCR for the simultaneous detection and differentiation of West Nile virus lineages 1 and 2, and of Usutu virus. J. Virol. Methods.

[21] Nikolay B., Weidmann M., Dupressoir A., Faye O., Boye C., Diallo M., Sall A. (2014). Development of a Usutu virus specific real-time reverse transcription PCR assay based on sequenced strains from Africa and Europe. J. Virol. Methods.

[22] Hönig V., Palus M., Kaspar T., Zemanova M., Majerova K., Hofmannova L., Papezik P., Sikutova S., Rettich F., Hubalek Z. (2019). Multiple Lineages of Usutu Virus (Flaviviridae, Flavivirus) in Blackbirds (Turdus merula) and Mosquitoes (Culex pipiens, Cx. modestus) in the Czech Republic (2016–2019). Microorganisms.

[23] Hall T.A. (1999). BioEdit: A user-friendly biological sequence alignment editor and analysis program for Windows 95/98/NT. Nucl. Acids. Symp. Ser..

[24] Tamura K., Stecher G., Kumar S. (2021). MEGA11: Molecular Evolutionary Genetics Analysis Version 11. Mol. Biol. Evol..

[25] Subramanian B., Gao S., Lercher M.J., Hu S., Chen W.-H. (2019). Evolview v3: A webserver for visualization, annotation, and management of phylogenetic trees. Nucleic Acids Res..

[26] Zecchin B., Fusaro A., Milani A., Schivo A., Ravagnan S., Ormelli S., Mavian C., Michelutti A., Toniolo F., Barzon L. (2021). The central role of Italy in the spatial spread of USUTU virus in Europe. Virus Evol..

[27] Gaibani P., Cavrini F., Gould E.A., Rossini G., Pierro A., Landini M.P., Sambri V. (2013). Comparative Genomic and Phylogenetic Analysis of the First Usutu Virus Isolate from a Human Patient Presenting with Neurological Symptoms. PLoS ONE.

[28] Kolodziejek J., Marinov M., Kiss B.J., Alexe V., Nowotny N. (2014). The Complete Sequence of a West Nile Virus Lineage 2 Strain Detected in a Hyalomma marginatum marginatum Tick Collected from a Song Thrush (Turdus philomelos) in Eastern Romania in 2013 Revealed Closest Genetic Relationship to Strain Volgograd 2007. PLoS ONE.

[29] Dinu S., Cotar A.I., Panculescu-Gatej I.R., Falcuta E., Prioteasa F.L., Sirbu A., Oprisan G., Badescu D., Reiter P., Ceianu C.S. (2015). West Nile virus circulation in south-eastern Romania, 2011 to 2013. Euro Surveill. Bull. Eur. Sur Les Mal. Transm. = Eur. Commun. Dis. Bull..

[30] Cotar A.I., Falcuta E., Prioteasa L.F., Dinu S., Ceianu C.S., Paz S. (2016). Transmission Dynamics of the West Nile Virus in Mosquito Vector Populations under the Influence of Weather Factors in the Danube Delta, Romania. EcoHealth.

[31] Tomazatos A., Jansen S., Pfister S., Török E., Maranda I., Horváth C., Keresztes L., Spînu M., Tannich E., Jöst H. (2019). Ecology of West Nile Virus in the Danube Delta, Romania: Phylogeography, Xenosurveillance and Mosquito Host-Feeding Patterns. Viruses.

[32] Tsai T.F., Popovici F., Cernescu C., Campbell G.L., Nedelcu N.I. (1998). West Nile encephalitis epidemic in southeastern Romania. Lancet.

[33] Sirbu A., Ceianu C.S., Panculescu-Gatej R.I., Vazquez A., Tenorio A., Rebreanu R., Niedrig M., Nicolescu G., Pistol A. (2011). Outbreak of West Nile virus infection in humans, Romania, July to October 2010. Euro Surveill. Bull. Eur. Sur Les Mal. Transm. = Eur. Commun. Dis. Bull..

[34] Popescu C.P., Florescu S.A., Cotar A.I., Badescu D., Ceianu C.S., Zaharia M., Tardei G., Codreanu D., Ceausu E., Ruta S.M. (2018). Re-emergence of severe West Nile virus neuroinvasive disease in humans in Romania, 2012 to 2017–implications for travel medicine. Travel Med. Infect. Dis..

[35] Savage H.M., Romanca C., Vladimirescu A., Tsai T.F., Ceianu C., Karabatsos N., Lanciotti R., Ungureanu A., Laiv L., Nicolescu G. (1999). Entomologic and avian investigations of an epidemic of West Nile fever in Romania in 1996, with serologic and molecular characterization of a virus isolate from mosquitoes. Am. J. Trop. Med. Hyg..

[36] Cotar A.I., Fălcuță E., Dinu S., Necula A., Bîrluțiu V., Ceianu C.S., Prioteasa F.L. (2018). West Nile virus lineage 2 in Romania, 2015–2016: Co-circulation and strain replacement. Parasites Vectors.

[37] Vilibic-Cavlek T., Savic V., Petrovic T., Toplak I., Barbic L., Petric D., Tabain I., Hrnjakovic-Cvjetkovic I., Bogdanic M., Klobucar A. (2019). Emerging Trends in the Epidemiology of West Nile and Usutu Virus Infections in Southern Europe. Front. Veter- Sci..

[38] Salgado R., Hawks S.A., Frere F., Vázquez A., Huang C.Y.-H., Duggal N.K. (2021). West Nile Virus Vaccination Protects against Usutu Virus Disease in Mice. Viruses.

[39] Wang H., Abbo S.R., Visser T.M., Westenberg M., Geertsema C., Fros J.J., Koenraadt C.J.M., Pijlman G.P. (2020). Competition between Usutu virus and West Nile virus during simultaneous and sequential infection of *Culex pipiens* mosquitoes. Emerg. Microbes Infect..

[40] European Centre for Disease Prevention and Control Transmission of West Nile Virus, July to December 2019–Table of Cases, 2019 Transmission Season. https://www.ecdc.europa.eu/en/publications-data/transmission-west-nile-virus-july-december-2019-table-cases-2019-transmission.

